# Supplementary material for: Phase Separation Phenomena in Lightly Cu-Doped A-Site-Ordered Quadruple Perovskite NdMn7O12
Source: Molecules. 2025 Nov 26;30(23):4561. doi: 10.3390/molecules30234561 (PMC12692892; doi:10.3390/molecules30234561)
Supplement: Supplementary file 1 [file molecules-30-04561-s001.zip › molecules-3984411-supplementary.pdf]

**Table S1.** Structure parameters of two monoclinic phases in $NdCu_{0.3}Mn_{6.7}O_{12}$.

Reliability factors, goodness-of-fit indicator,and Durbin-Watson statistic
Rwp = 4.07  Rp = 2.55  RR = 18.08  Re = 0.77  S = 5.3150  d1 = 0.6809  d2 = 0.9013
NdMn6.7Cu0.3O12 I2/m-1
RI = 4.54  RF = 3.86  E(SCIO) = 19761.5
NdMn6.7Cu0.3O12 I2/m-2
RI = 4.62  RF = 3.98  E(SCIO) = 22953.3

Lattice parameters (Angstrom or degree) and unit-cell volume (Angstrom**3) in NdMn6.7Cu0.3O12 I2/m-1

| a | b | c | alpha | beta | gamma | V |
|---|---|---|---|---|---|---|
| 7.47143 | 7.36828 | 7.46210 | 90.0000 | 90.9929 | 90.0000 | 410.7387 |
| 0.00002 | 0.00002 | 0.00002 | - | 0.0003 | - | 0.0019 |

Structure parameters, g, x, y, z, B/Angstrom**2, and U/Angstrom**2, in NdMn6.7Cu0.3O12 I2/m-1
100*B/nm**2  100*U/nm**2

| | neq | * g | = n | x | y | z | B | U |
|---|---|---|---|---|---|---|---|---|
| Nd | 2 | 1.0000 | 2.0000 | 0.00000 | 0.00000 | 0.00000 | 0.445 | 0.00564 |
| | - | - | - | - | - | - | 0.051 | 0.00064 |
| Mn1 | 2 | 1.0000 | 2.0000 | 0.00000 | 0.50000 | 0.00000 | 0.413 | 0.00524 |
| | - | - | - | - | - | - | 0.115 | 0.00146 |
| Mn2 | 2 | 1.0000 | 2.0000 | 0.50000 | 0.00000 | 0.00000 | 0.312 | 0.00395 |
| | - | - | - | - | - | - | 0.128 | 0.00162 |
| Mn3 | 2 | 1.0000 | 2.0000 | 0.50000 | 0.50000 | 0.00000 | 0.444 | 0.00563 |
| | - | - | - | - | - | - | 0.132 | 0.00167 |
| Mn4 | 4 | 1.0000 | 4.0000 | 0.25000 | 0.25000 | 0.25000 | 0.420 | 0.00532 |
| | - | - | - | - | - | - | 0.053 | 0.00067 |
| Mn5 | 4 | 1.0000 | 4.0000 | 0.25000 | 0.25000 | 0.75000 | 0.395 | 0.00500 |
| | - | - | - | - | - | - | 0.053 | 0.00068 |
| O1a | 4 | 1.0000 | 4.0000 | 0.17055 | 0.00000 | 0.31192 | 0.800 | 0.01013 |
| | - | - | - | 0.00187 | - | 0.00209 | - | - |
| O2a | 4 | 1.0000 | 4.0000 | 0.18201 | 0.00000 | 0.67272 | 0.800 | 0.01013 |
| | - | - | - | 0.00190 | - | 0.00225 | - | - |
| O3a | 8 | 1.0000 | 8.0000 | 0.01293 | 0.31171 | 0.18638 | 0.800 | 0.01013 |
| | - | - | - | 0.00138 | 0.00146 | 0.00148 | - | - |
| O4a | 8 | 1.0000 | 8.0000 | 0.30087 | 0.16874 | -0.00876 | 0.800 | 0.01013 |
| | - | - | - | 0.00132 | 0.00156 | 0.00163 | - | - |

Lattice parameters (Angstrom or degree) and unit-cell volume (Angstrom**3) in NdMn6.7Cu0.3O12 I2/m-2

| a | b | c | alpha | beta | gamma | V |
|---|---|---|---|---|---|---|
| 7.46596 | 7.37257 | 7.45756 | 90.0000 | 90.9328 | 90.0000 | 410.4340 |
| 0.00003 | 0.00003 | 0.00003 | - | 0.0004 | - | 0.0029 |

Structure parameters, g, x, y, z, B/Angstrom**2, and U/Angstrom**2, in NdMn6.7Cu0.3O12 I2/m-2
100*B/nm**2  100*U/nm**2

| | neq | * g | = | n | x | y | z | B | U |
|---|---|---|---|---|---|---|---|---|---|
| Ndb | 2 | 1.0000 | | 2.0000 | 0.00000 | 0.00000 | 0.00000 | 0.310 | 0.00392 |
| | - | - | - | - | - | - | | 0.049 | 0.00062 |
| Mn1b | 2 | 1.0000 | | 2.0000 | 0.00000 | 0.50000 | 0.00000 | 0.370 | 0.00469 |
| | - | - | - | - | - | - | | 0.116 | 0.00147 |
| Mn2b | 2 | 1.0000 | | 2.0000 | 0.50000 | 0.00000 | 0.00000 | 0.863 | 0.01094 |
| | - | - | - | - | - | - | | 0.162 | 0.00205 |
| Mn3b | 2 | 1.0000 | | 2.0000 | 0.50000 | 0.50000 | 0.00000 | 0.980 | 0.01242 |
| | - | - | - | - | - | - | | 0.167 | 0.00212 |
| Mn4b | 4 | 1.0000 | | 4.0000 | 0.25000 | 0.25000 | 0.25000 | 0.279 | 0.00354 |
| | - | - | - | - | - | - | - | - | |
| Mn5b | 4 | 1.0000 | | 4.0000 | 0.25000 | 0.25000 | 0.75000 | 0.199 | 0.00252 |
| | - | - | - | - | - | - | - | - | |
| O1b | 4 | 1.0000 | | 4.0000 | 0.15024 | 0.00000 | 0.30569 | 0.800 | 0.01013 |
| | - | - | - | | 0.00206 | - | 0.00224 | - | - |
| O2b | 4 | 1.0000 | | 4.0000 | 0.16205 | 0.00000 | 0.68585 | 0.800 | 0.01013 |
| | - | - | - | | 0.00217 | - | 0.00238 | - | - |
| O3b | 8 | 1.0000 | | 8.0000 | 0.00890 | 0.30454 | 0.18153 | 0.800 | 0.01013 |
| | - | - | - | | 0.00155 | 0.00163 | 0.00158 | - | - |
| O4b | 8 | 1.0000 | | 8.0000 | 0.30891 | 0.17293 | -0.00534 | 0.800 | 0.01013 |
| | - | - | - | | 0.00151 | 0.00180 | 0.00175 | - | - |

Mass fractions of compounds contained in the sample

| | |
|---|---|
| NdMn6.7Cu0.3O12 I2/m-1 | 0.4987 |
| NdMn6.7Cu0.3O12 I2/m-2 | 0.5013 |

```
INTERATOMIC DISTANCE IN ANGSTROMS  NdMn6.7Cu0.3O12  I2/m-1
   1    ( 1,    0) (11,    0)              Nd       ( 0.0000,  0.0000,  0.0000);   O4a      ( 0.3009,  0.1687, -0.0088)
                                      2.5707 +OR- 0.0100 ( 0.0100)
   2    ( 1,    0) (11,    2)              Nd       ( 0.0000,  0.0000,  0.0000);   O4a      (-0.3009, -0.1687,  0.0088)
                                      2.5707 +OR- 0.0100 ( 0.0100)
   3    ( 1,    0) (11,    4)              Nd       ( 0.0000,  0.0000,  0.0000);   O4a      (-0.3009,  0.1687,  0.0088)
                                      2.5707 +OR- 0.0100 ( 0.0100)
   4    ( 1,    0) (11,    6)              Nd       ( 0.0000,  0.0000,  0.0000);   O4a      ( 0.3009, -0.1687, -0.0088)
                                      2.5707 +OR- 0.0100 ( 0.0100)
   5    ( 1,    0) ( 8,    0)              Nd       ( 0.0000,  0.0000,  0.0000);   O1a      ( 0.1705,  0.0000,  0.3119)
                                      2.6341 +OR- 0.0165 ( 0.0165)
   6    ( 1,    0) ( 8,    2)              Nd       ( 0.0000,  0.0000,  0.0000);   O1a      (-0.1705,  0.0000, -0.3119)
                                      2.6341 +OR- 0.0165 ( 0.0165)
   7    ( 1,    0) (10,    0)              Nd       ( 0.0000,  0.0000,  0.0000);   O3a      ( 0.0129,  0.3117,  0.1864)
                                      2.6859 +OR- 0.0108 ( 0.0108)
   8    ( 1,    0) (10,    2)              Nd       ( 0.0000,  0.0000,  0.0000);   O3a      (-0.0129, -0.3117, -0.1864)
                                      2.6859 +OR- 0.0108 ( 0.0108)
   9    ( 1,    0) (10,    4)              Nd       ( 0.0000,  0.0000,  0.0000);   O3a      (-0.0129,  0.3117, -0.1864)
                                      2.6859 +OR- 0.0108 ( 0.0108)
  10    ( 1,    0) (10,    6)              Nd       ( 0.0000,  0.0000,  0.0000);   O3a      ( 0.0129, -0.3117,  0.1864)
                                      2.6859 +OR- 0.0108 ( 0.0108)
  11    ( 1,    0) ( 9,11000)              Nd       ( 0.0000,  0.0000,  0.0000);   O2a      ( 0.1820,  0.0000, -0.3273)
                                      2.8158 +OR- 0.0142 ( 0.0142)
  12    ( 1,    0) ( 9,23002)              Nd       ( 0.0000,  0.0000,  0.0000);   O2a      (-0.1820,  0.0000,  0.3273)
                                      2.8158 +OR- 0.0142 ( 0.0142)
---------------------------------------------------------------------------------------------------------------------------
  25    ( 3,    0) (10,    0)              Mn1      ( 0.0000,  0.5000,  0.0000);   O3a      ( 0.0129,  0.3117,  0.1864)
                                      1.9657 +OR- 0.0110 ( 0.0110)
  26    ( 3,    0) (10,    4)              Mn1      ( 0.0000,  0.5000,  0.0000);   O3a      (-0.0129,  0.3117, -0.1864)
                                      1.9657 +OR- 0.0110 ( 0.0110)
  27    ( 3,    0) (10,21002)              Mn1      ( 0.0000,  0.5000,  0.0000);   O3a      (-0.0129,  0.6883, -0.1864)
                                      1.9657 +OR- 0.0110 ( 0.0110)
  28    ( 3,    0) (10,21006)              Mn1      ( 0.0000,  0.5000,  0.0000);   O3a      ( 0.0129,  0.6883,  0.1864)
                                      1.9657 +OR- 0.0110 ( 0.0110)
  29    ( 3,    0) ( 9,    3)              Mn1      ( 0.0000,  0.5000,  0.0000);   O2a      ( 0.3180,  0.5000, -0.1727)
                                      2.7224 +OR- 0.0126 ( 0.0126)
  30    ( 3,    0) ( 9,15001)              Mn1      ( 0.0000,  0.5000,  0.0000);   O2a      (-0.3180,  0.5000,  0.1727)
                                      2.7224 +OR- 0.0126 ( 0.0126)
  31    ( 3,    0) ( 8,    3)              Mn1      ( 0.0000,  0.5000,  0.0000);   O1a      ( 0.3295,  0.5000,  0.1881)
                                      2.8123 +OR- 0.0157 ( 0.0157)
  32    ( 3,    0) ( 8,15001)              Mn1      ( 0.0000,  0.5000,  0.0000);   O1a      (-0.3295,  0.5000, -0.1881)
                                      2.8123 +OR- 0.0157 ( 0.0157)
---------------------------------------------------------------------------------------------------------------------------
  33    ( 4,    0) (11,    0)              Mn2      ( 0.5000,  0.0000,  0.0000);   O4a      ( 0.3009,  0.1687, -0.0088)
                                      1.9391 +OR- 0.0108 ( 0.0108)
  34    ( 4,    0) (11,    6)              Mn2      ( 0.5000,  0.0000,  0.0000);   O4a      ( 0.3009, -0.1687, -0.0088)
                                      1.9391 +OR- 0.0108 ( 0.0108)
  35    ( 4,    0) (11,20002)              Mn2      ( 0.5000,  0.0000,  0.0000);   O4a      ( 0.6991, -0.1687,  0.0088)
                                      1.9391 +OR- 0.0108 ( 0.0108)
  36    ( 4,    0) (11,20004)              Mn2      ( 0.5000,  0.0000,  0.0000);   O4a      ( 0.6991,  0.1687,  0.0088)
                                      1.9391 +OR- 0.0108 ( 0.0108)
  37    ( 4,    0) (10,    3)              Mn2      ( 0.5000,  0.0000,  0.0000);   O3a      ( 0.4871,  0.1883,  0.3136)
                                      2.7237 +OR- 0.0108 ( 0.0108)
  38    ( 4,    0) (10, 5005)              Mn2      ( 0.5000,  0.0000,  0.0000);   O3a      ( 0.4871, -0.1883,  0.3136)
                                      2.7237 +OR- 0.0108 ( 0.0108)
  39    ( 4,    0) (10,11007)              Mn2      ( 0.5000,  0.0000,  0.0000);   O3a      ( 0.5129,  0.1883, -0.3136)
                                      2.7237 +OR- 0.0108 ( 0.0108)
  40    ( 4,    0) (10,17001)              Mn2      ( 0.5000,  0.0000,  0.0000);   O3a      ( 0.5129, -0.1883, -0.3136)
                                      2.7237 +OR- 0.0108 ( 0.0108)
---------------------------------------------------------------------------------------------------------------------------
  41    ( 5,    0) ( 9,    3)              Mn3      ( 0.5000,  0.5000,  0.0000);   O2a      ( 0.3180,  0.5000, -0.1727)
                                      1.8574 +OR- 0.0176 ( 0.0176)
  42    ( 5,    0) ( 9,11001)              Mn3      ( 0.5000,  0.5000,  0.0000);   O2a      ( 0.6820,  0.5000,  0.1727)
                                      1.8574 +OR- 0.0176 ( 0.0176)
  43    ( 5,    0) ( 8,    3)              Mn3      ( 0.5000,  0.5000,  0.0000);   O1a      ( 0.3295,  0.5000,  0.1881)
                                      1.9119 +OR- 0.0131 ( 0.0131)
  44    ( 5,    0) ( 8,11001)              Mn3      ( 0.5000,  0.5000,  0.0000);   O1a      ( 0.6705,  0.5000, -0.1881)
                                      1.9119 +OR- 0.0131 ( 0.0131)
  45    ( 5,    0) (11,    0)              Mn3      ( 0.5000,  0.5000,  0.0000);   O4a      ( 0.3009,  0.1687, -0.0088)
                                      2.8586 +OR- 0.0109 ( 0.0109)
  46    ( 5,    0) (11,20004)              Mn3      ( 0.5000,  0.5000,  0.0000);   O4a      ( 0.6991,  0.1687,  0.0088)
                                      2.8586 +OR- 0.0109 ( 0.0109)
  47    ( 5,    0) (11,21006)              Mn3      ( 0.5000,  0.5000,  0.0000);   O4a      ( 0.3009,  0.8313, -0.0088)
                                      2.8586 +OR- 0.0109 ( 0.0109)
  48    ( 5,    0) (11,22002)              Mn3      ( 0.5000,  0.5000,  0.0000);   O4a      ( 0.6991,  0.8313,  0.0088)
                                      2.8586 +OR- 0.0109 ( 0.0109)
---------------------------------------------------------------------------------------------------------------------------
  49    ( 6,    0) (10,    0)              Mn4      ( 0.2500,  0.2500,  0.2500);   O3a      ( 0.0129,  0.3117,  0.1864)
                                      1.8816 +OR- 0.0111 ( 0.0111)
  50    ( 6,    0) (10,    3)              Mn4      ( 0.2500,  0.2500,  0.2500);   O3a      ( 0.4871,  0.1883,  0.3136)
```

```
                                   1.8816 +OR- 0.0111 ( 0.0111)
  51   ( 6,   0) ( 8,   0)            Mn4    ( 0.2500,  0.2500,  0.2500);   O1a    ( 0.1705,  0.0000,  0.3119)
                                   1.9921 +OR- 0.0049 ( 0.0049)
  52   ( 6,   0) ( 8,   3)            Mn4    ( 0.2500,  0.2500,  0.2500);   O1a    ( 0.3295,  0.5000,  0.1881)
                                   1.9921 +OR- 0.0049 ( 0.0049)
  53   ( 6,   0) (11,   0)            Mn4    ( 0.2500,  0.2500,  0.2500);   O4a    ( 0.3009,  0.1687, -0.0088)
                                   2.0632 +OR- 0.0113 ( 0.0113)
  54   ( 6,   0) (11,   3)            Mn4    ( 0.2500,  0.2500,  0.2500);   O4a    ( 0.1991,  0.3313,  0.5088)
                                   2.0632 +OR- 0.0113 ( 0.0113)
------------------------------------------------------------------------------------------------------------------------
  55   ( 7,   0) (11,   3)            Mn5    ( 0.2500,  0.2500,  0.7500);   O4a    ( 0.1991,  0.3313,  0.5088)
                                   1.9287 +OR- 0.0127 ( 0.0127)
  56   ( 7,   0) (11,23000)           Mn5    ( 0.2500,  0.2500,  0.7500);   O4a    ( 0.3009,  0.1687,  0.9912)
                                   1.9287 +OR- 0.0127 ( 0.0127)
  57   ( 7,   0) ( 9,   0)            Mn5    ( 0.2500,  0.2500,  0.7500);   O2a    ( 0.1820,  0.0000,  0.6727)
                                   1.9934 +OR- 0.0068 ( 0.0068)
  58   ( 7,   0) ( 9,23003)           Mn5    ( 0.2500,  0.2500,  0.7500);   O2a    ( 0.3180,  0.5000,  0.8273)
                                   1.9934 +OR- 0.0068 ( 0.0068)
  59   ( 7,   0) (10,   7)            Mn5    ( 0.2500,  0.2500,  0.7500);   O3a    ( 0.5129,  0.1883,  0.6864)
                                   2.0793 +OR- 0.0095 ( 0.0095)
  60   ( 7,   0) (10,23004)           Mn5    ( 0.2500,  0.2500,  0.7500);   O3a    (-0.0129,  0.3117,  0.8136)
                                   2.0793 +OR- 0.0095 ( 0.0095)
------------------------------------------------------------------------------------------------------------------------


INTERATOMIC DISTANCE IN ANGSTROMS   NdMn6.7Cu0.3O12  I2/m-2
   1   ( 1,   0) ( 7,   0)            Ndb    ( 0.0000,  0.0000,  0.0000);   O1b    ( 0.1502,  0.0000,  0.3057)
                                   2.5243 +OR- 0.0178 ( 0.0178)
   2   ( 1,   0) ( 7,   2)            Ndb    ( 0.0000,  0.0000,  0.0000);   O1b    (-0.1502,  0.0000, -0.3057)
                                   2.5243 +OR- 0.0178 ( 0.0178)
   3   ( 1,   0) ( 9,   0)            Ndb    ( 0.0000,  0.0000,  0.0000);   O3b    ( 0.0089,  0.3045,  0.1815)
                                   2.6221 +OR- 0.0116 ( 0.0116)
   4   ( 1,   0) ( 9,   2)            Ndb    ( 0.0000,  0.0000,  0.0000);   O3b    (-0.0089, -0.3045, -0.1815)
                                   2.6221 +OR- 0.0116 ( 0.0116)
   5   ( 1,   0) ( 9,   4)            Ndb    ( 0.0000,  0.0000,  0.0000);   O3b    (-0.0089,  0.3045, -0.1815)
                                   2.6221 +OR- 0.0116 ( 0.0116)
   6   ( 1,   0) ( 9,   6)            Ndb    ( 0.0000,  0.0000,  0.0000);   O3b    ( 0.0089, -0.3045,  0.1815)
                                   2.6221 +OR- 0.0116 ( 0.0116)
   7   ( 1,   0) (10,   0)            Ndb    ( 0.0000,  0.0000,  0.0000);   O4b    ( 0.3089,  0.1729, -0.0053)
                                   2.6361 +OR- 0.0112 ( 0.0112)
   8   ( 1,   0) (10,   2)            Ndb    ( 0.0000,  0.0000,  0.0000);   O4b    (-0.3089, -0.1729,  0.0053)
                                   2.6361 +OR- 0.0112 ( 0.0112)
   9   ( 1,   0) (10,   4)            Ndb    ( 0.0000,  0.0000,  0.0000);   O4b    (-0.3089,  0.1729,  0.0053)
                                   2.6361 +OR- 0.0112 ( 0.0112)
  10   ( 1,   0) (10,   6)            Ndb    ( 0.0000,  0.0000,  0.0000);   O4b    ( 0.3089, -0.1729, -0.0053)
                                   2.6361 +OR- 0.0112 ( 0.0112)
  11   ( 1,   0) ( 8,11000)           Ndb    ( 0.0000,  0.0000,  0.0000);   O2b    ( 0.1620,  0.0000, -0.3142)
                                   2.6542 +OR- 0.0152 ( 0.0152)
  12   ( 1,   0) ( 8,23002)           Ndb    ( 0.0000,  0.0000,  0.0000);   O2b    (-0.1620,  0.0000,  0.3142)
                                   2.6542 +OR- 0.0152 ( 0.0152)
------------------------------------------------------------------------------------------------------------------------
  13   ( 2,   0) ( 9,   0)            Mn1b   ( 0.0000,  0.5000,  0.0000);   O3b    ( 0.0089,  0.3045,  0.1815)
                                   1.9776 +OR- 0.0124 ( 0.0124)
  14   ( 2,   0) ( 9,   4)            Mn1b   ( 0.0000,  0.5000,  0.0000);   O3b    (-0.0089,  0.3045, -0.1815)
                                   1.9776 +OR- 0.0124 ( 0.0124)
  15   ( 2,   0) ( 9,21002)           Mn1b   ( 0.0000,  0.5000,  0.0000);   O3b    (-0.0089,  0.6955, -0.1815)
                                   1.9776 +OR- 0.0124 ( 0.0124)
  16   ( 2,   0) ( 9,21006)           Mn1b   ( 0.0000,  0.5000,  0.0000);   O3b    ( 0.0089,  0.6955,  0.1815)
                                   1.9776 +OR- 0.0124 ( 0.0124)
  17   ( 2,   0) ( 8,   3)            Mn1b   ( 0.0000,  0.5000,  0.0000);   O2b    ( 0.3380,  0.5000, -0.1858)
                                   2.8984 +OR- 0.0141 ( 0.0141)
  18   ( 2,   0) ( 8,15001)           Mn1b   ( 0.0000,  0.5000,  0.0000);   O2b    (-0.3380,  0.5000,  0.1858)
                                   2.8984 +OR- 0.0141 ( 0.0141)
  19   ( 2,   0) ( 7,   3)            Mn1b   ( 0.0000,  0.5000,  0.0000);   O1b    ( 0.3498,  0.5000,  0.1943)
                                   2.9657 +OR- 0.0172 ( 0.0172)
  20   ( 2,   0) ( 7,15001)           Mn1b   ( 0.0000,  0.5000,  0.0000);   O1b    (-0.3498,  0.5000, -0.1943)
                                   2.9657 +OR- 0.0172 ( 0.0172)
------------------------------------------------------------------------------------------------------------------------
  21   ( 3,   0) (10,   0)            Mn2b   ( 0.5000,  0.0000,  0.0000);   O4b    ( 0.3089,  0.1729, -0.0053)
                                   1.9133 +OR- 0.0127 ( 0.0127)
  22   ( 3,   0) (10,   6)            Mn2b   ( 0.5000,  0.0000,  0.0000);   O4b    ( 0.3089, -0.1729, -0.0053)
                                   1.9133 +OR- 0.0127 ( 0.0127)
  23   ( 3,   0) (10,20002)           Mn2b   ( 0.5000,  0.0000,  0.0000);   O4b    ( 0.6911, -0.1729,  0.0053)
                                   1.9133 +OR- 0.0127 ( 0.0127)
  24   ( 3,   0) (10,20004)           Mn2b   ( 0.5000,  0.0000,  0.0000);   O4b    ( 0.6911,  0.1729,  0.0053)
                                   1.9133 +OR- 0.0127 ( 0.0127)
  25   ( 3,   0) ( 9,   3)            Mn2b   ( 0.5000,  0.0000,  0.0000);   O3b    ( 0.4911,  0.1955,  0.3185)
                                   2.7797 +OR- 0.0114 ( 0.0114)
  26   ( 3,   0) ( 9, 5005)           Mn2b   ( 0.5000,  0.0000,  0.0000);   O3b    ( 0.4911, -0.1955,  0.3185)
                                   2.7797 +OR- 0.0114 ( 0.0114)
```

```
  27   ( 3,   0) ( 9,11007)          Mn2b   ( 0.5000,  0.0000,  0.0000);   O3b    ( 0.5089,  0.1955, -0.3185)
                                  2.7797 +OR- 0.0114 ( 0.0114)
  28   ( 3,   0) ( 9,17001)          Mn2b   ( 0.5000,  0.0000,  0.0000);   O3b    ( 0.5089, -0.1955, -0.3185)
                                  2.7797 +OR- 0.0114 ( 0.0114)
-----------------------------------------------------------------------------------------------------------
  29   ( 4,   0) ( 8,    3)          Mn3b   ( 0.5000,  0.5000,  0.0000);   O2b    ( 0.3380,  0.5000, -0.1858)
                                  1.8248 +OR- 0.0194 ( 0.0194)
  30   ( 4,   0) ( 8,11001)          Mn3b   ( 0.5000,  0.5000,  0.0000);   O2b    ( 0.6620,  0.5000,  0.1858)
                                  1.8248 +OR- 0.0194 ( 0.0194)
  31   ( 4,   0) ( 7,    3)          Mn3b   ( 0.5000,  0.5000,  0.0000);   O1b    ( 0.3498,  0.5000,  0.1943)
                                  1.8468 +OR- 0.0142 ( 0.0142)
  32   ( 4,   0) ( 7,11001)          Mn3b   ( 0.5000,  0.5000,  0.0000);   O1b    ( 0.6502,  0.5000, -0.1943)
                                  1.8468 +OR- 0.0142 ( 0.0142)
  33   ( 4,   0) (10,    0)          Mn3b   ( 0.5000,  0.5000,  0.0000);   O4b    ( 0.3089,  0.1729, -0.0053)
                                  2.8017 +OR- 0.0123 ( 0.0123)
  34   ( 4,   0) (10,20004)          Mn3b   ( 0.5000,  0.5000,  0.0000);   O4b    ( 0.6911,  0.1729,  0.0053)
                                  2.8017 +OR- 0.0123 ( 0.0123)
  35   ( 4,   0) (10,21006)          Mn3b   ( 0.5000,  0.5000,  0.0000);   O4b    ( 0.3089,  0.8271, -0.0053)
                                  2.8017 +OR- 0.0123 ( 0.0123)
  36   ( 4,   0) (10,22002)          Mn3b   ( 0.5000,  0.5000,  0.0000);   O4b    ( 0.6911,  0.8271,  0.0053)
                                  2.8017 +OR- 0.0123 ( 0.0123)
-----------------------------------------------------------------------------------------------------------
  37   ( 5,   0) ( 9,    0)          Mn4b   ( 0.2500,  0.2500,  0.2500);   O3b    ( 0.0089,  0.3045,  0.1815)
                                  1.9059 +OR- 0.0125 ( 0.0125)
  38   ( 5,   0) ( 9,    3)          Mn4b   ( 0.2500,  0.2500,  0.2500);   O3b    ( 0.4911,  0.1955,  0.3185)
                                  1.9059 +OR- 0.0125 ( 0.0125)
  39   ( 5,   0) ( 7,    0)          Mn4b   ( 0.2500,  0.2500,  0.2500);   O1b    ( 0.1502,  0.0000,  0.3057)
                                  2.0334 +OR- 0.0059 ( 0.0059)
  40   ( 5,   0) ( 7,    3)          Mn4b   ( 0.2500,  0.2500,  0.2500);   O1b    ( 0.3498,  0.5000,  0.1943)
                                  2.0334 +OR- 0.0059 ( 0.0059)
  41   ( 5,   0) (10,    0)          Mn4b   ( 0.2500,  0.2500,  0.2500);   O4b    ( 0.3089,  0.1729, -0.0053)
                                  2.0419 +OR- 0.0119 ( 0.0119)
  42   ( 5,   0) (10,    3)          Mn4b   ( 0.2500,  0.2500,  0.2500);   O4b    ( 0.1911,  0.3271,  0.5053)
                                  2.0419 +OR- 0.0119 ( 0.0119)
-----------------------------------------------------------------------------------------------------------
  43   ( 6,   0) (10,    3)          Mn5b   ( 0.2500,  0.2500,  0.7500);   O4b    ( 0.1911,  0.3271,  0.5053)
                                  1.9543 +OR- 0.0141 ( 0.0141)
  44   ( 6,   0) (10,23000)          Mn5b   ( 0.2500,  0.2500,  0.7500);   O4b    ( 0.3089,  0.1729,  0.9947)
                                  1.9543 +OR- 0.0141 ( 0.0141)
  45   ( 6,   0) ( 8,    0)          Mn5b   ( 0.2500,  0.2500,  0.7500);   O2b    ( 0.1620,  0.0000,  0.6858)
                                  2.0117 +OR- 0.0076 ( 0.0076)
  46   ( 6,   0) ( 8,23003)          Mn5b   ( 0.2500,  0.2500,  0.7500);   O2b    ( 0.3380,  0.5000,  0.8142)
                                  2.0117 +OR- 0.0076 ( 0.0076)
  47   ( 6,   0) ( 9,    7)          Mn5b   ( 0.2500,  0.2500,  0.7500);   O3b    ( 0.5089,  0.1955,  0.6815)
                                  2.0471 +OR- 0.0106 ( 0.0106)
  48   ( 6,   0) ( 9,23004)          Mn5b   ( 0.2500,  0.2500,  0.7500);   O3b    (-0.0089,  0.3045,  0.8185)
                                  2.0471 +OR- 0.0106 ( 0.0106)
-----------------------------------------------------------------------------------------------------------
```
